# *Toxoplasma gondii* among pregnant women attending antenatal care in a district hospital in Ghana

**DOI:** 10.1002/puh2.82

**Published:** 2023-05-25

**Authors:** Seth Offei Addo, Thelma Ohene Asare, Christina Arthur, Kenneth Amoateng, Kelvin Addo, John Asiedu Larbi

**Affiliations:** ^1^ Department of Parasitology Noguchi Memorial Institute for Medical Research, University of Ghana Accra Ghana; ^2^ Department of Theoretical and Applied Biology Kwame Nkrumah University of Science and Technology Kumasi Ghana

**Keywords:** antenatal care, congenital infection, pregnancy outcome, pregnant women, zoonosis

## Abstract

**Background:**

Toxoplasmosis is a zoonotic disease that is asymptomatic in many but can spread through the placenta during pregnancy causing miscarriage, stillbirth, intrauterine death, and neurologic impairments. This study reports on the proportion and risk factors associated with the seropositivity of *Toxoplasma gondii* among pregnant women visiting the antenatal care unit of the Nkawie‐Toase Government Hospital, Ghana.

**Methods:**

This was a cross‐sectional study among pregnant women attending antenatal care at a district hospital in Ghana. The participants were selected randomly for the study. A structured questionnaire was used to collect information and data on possible risk factors associated with toxoplasmosis. Serum samples from the participants were screened for exposure to *T. gondii* using a commercial ELISA kit. The associations of the risk factors with seropositivity were tested using logistic regression.

**Results:**

There were 84 women who participated in this study. The mean age of the sample was 26.96 ± 5.67 years; 64.29% had basic education, 21.43% were traders and 14.29% were farmers. Forty‐seven women (55.95%) tested seropositive for *T. gondii*, 44 (52.38%) for IgG and 3 (3.57%) for IgM. Women involved as traders (OR 18, 95% CI 2.74–118.39, *p* = 0.003) and farmers (OR 6.75, 95% CI 1.16–39.20, *p* = 0.033) had significantly higher risk of testing seropositive.

**Conclusion:**

High proportions of pregnant women in this study were found to be exposed to *T. gondii*. To prevent negative pregnancy outcomes, pregnant women attending antenatal care be educated and routinely screened for *T. gondii* infection.

## INTRODUCTION

Toxoplasmosis is a zoonotic disease, caused by *Toxoplasma gondii*, infecting nearly all warm‐blooded vertebrates, including humans [1, 2]. Felids, such as household cats, are the parasite's only reported definitive hosts [3, 4, 5, 6]. This parasite infects humans mostly through the ingestion of food or water contaminated with oocysts shed by cats, or through the consumption of undercooked or raw meat harbouring tissue cysts [7]. Tachyzoites can be found in blood products, tissue transplants, and unpasteurized milk [8]. Several risk factors, including education level, contact with cats, contact with soil, parity and consumption of raw fruits and vegetables, influence *T. gondii* transmission [9, 10].

In most people, *T. gondii* infection is asymptomatic and self‐limiting, but it can occasionally cause minor disease [11]. The parasite's capacity to pass difficult biological barriers in infected host tissues, such as the brain, eye and placenta, is a key aspect of its pathogenesis [12]. Toxoplasmosis in foetuses and immunocompromised individuals results in considerable morbidity and mortality [13]. A mother infected with *T. gondii* in the first trimester stands a 10%–15% risk of transmitting the infection to the foetus with serious consequences [14]. However, mothers have up to 68% chance of infecting their unborn during the second or third trimesters, with less severe consequences [14]. When left undiagnosed or untreated, *T. gondii* infections during pregnancy can result in congenital toxoplasmosis which can be asymptomatic or severe with symptoms, including seizures, jaundice, loss of vision, delays in development and thrombocytopenia [15, 16].

In Africa, the seropositivity for toxoplasma infection is 18.7%–46.0% in Mozambique [17, 18], 20.3% in Burkina Faso [19], 40.8% in Nigeria [20] and 30.0% in Benin [21]. In Ghana, the seropositivity among pregnant women ranged from 51.2% to 92.5% in Accra [10, 22] and 71.8% in Ashanti Region [23]. In Kumasi, the seropositivity was 45.1% among women and 22.7% among infants [24], and 57.3% by enzyme‐linked immune assay (ELISA) and 21.5% by rapid diagnostic test (RDT) [25] among pregnant women in their first trimester. In the Greater Accra region, the seropositivity was 39.5% in samples from the umbilical cord, 37.6% among mothers and 57.5% among infants [26].

Anti‐*T. gondii* IgM antibody occurs during acute primary infection with the parasite and the titres start to drop during the following few months and become undetectable within a year [27]. A few weeks after the first infection, anti‐*T. gondii* IgG are produced reaching their peak in 1 to 2 months and remain detectable life long [28]. A polymerase chain reaction confirms current infections [29]. Early detection of the infection is crucial in preventing negative consequences on the pregnancy and her unborn child [25]. We describe the seropositivity of *T. gondii* among pregnant women attending antenatal care at a general hospital in Atwima‐Nwabiagya District, Ghana.

## METHODS

### Study design, setting and sample size

This study was a cross‐sectional study among pregnant women attending the antenatal unit of the Nkawie‐Toase Government Hospital, Atwima‐Nwabiagya District, Ghana in 2020. The district has a population of 197,874 with most inhabitants living in rural communities [30]. In Ghana, screening for the TORCH group of infections (toxoplasmosis, rubella, cytomegalovirus and herpes simplex virus) is not mandatory, and as such, pregnant women are not routinely screened for toxoplasmosis during antenatal visits.

The sample size was calculated for proportions with an estimated seropositivity of 92.5%; the sample size was 107. Pregnant women who did not consent to the study or were declared medically unfit by the attending physician were excluded.

### Data collection

The pregnant women attending the antenatal clinic were through simple random sampling. The collection of blood samples was undertaken following standard procedure [31]. Questionnaires were administered to obtain demographic information and the risk factors for *T. gondii* infections. The variables included the ages of participants, educational level, occupation and ownership of cats. The data were collected over a 6‐month period in 2020.

### Serological analysis

The seropositivity of *T. gondii* was tested for IgM and IgG using a commercial ELISA kit (Fortress Diagnostics, Antrim BT41 1QS, United Kingdom) at the Central Laboratory, Kwame Nkrumah University of Science and Technology. Using a microplate reader, the ELISA plates were read at a wavelength of 450 nm. The qualitative results were interpreted against standard reference values.

### Data analysis

The data were entered in Microsoft Excel 2019 and analysed using STATA version 13. Fisher's exact was used to describe the association between *T. gondii* positivity and the demographic factors. Univariate logistic regression was also used to determine the odds ratio for the examined factors, such as age, occupation, educational level and cat ownership. The significance level was set at *p* < 0.05.

### Ethical considerations

This research was approved by the Committee on Human Research, Publications, and Ethics (CHRPE) at the Kwame Nkrumah University of Science and Technology, School of Medical Sciences and Komfo Anokye Teaching Hospital (Ref: CHRPE/AP/062/20). Approval was also sought from the administration of the Nkawie‐Toase Government Hospital and all relevant authorities before commencing the study.

## RESULTS

Eighty‐four pregnant women participated in the study with a response rate of 78.50%. The mean age of the participants was 26.96 ± 5.67 years. Most of the pregnant women were in their third trimester (36.91%) and had no history of miscarriage or stillbirth (76.19%). The majority (69.05%) owned a cat. The basic socio‐demographic details of the participants are shown in Table 1.

**TABLE 1:** Socio‐demographic characteristics of pregnant women attending antenatal clinic tested for *Toxoplasma* serology at Nkawie‐Toase Government Hospital, Atwima‐Nwabiagya District, Ghana, 2020.

Variables	Frequency	%
**Seropositivity**	**47**	**55.95**
IgM	3	3.57
IgG	44	52.38
**Age (years)**		
<20	6	7.14
20–39	74	88.1
≥40	4	4.76
**Levels of education**		
None	12	14.29
Basic	54	64.29
Secondary	17	20.24
Tertiary	1	1.19
**Occupation**		
Trader	18	21.43
Farmer	12	14.29
Housewife	8	9.52
Hairdresser	5	5.95
Seamstress	5	5.95
Student	4	4.76
Teacher	3	3.57
Caterer	3	3.57
Civil servant	1	1.19
Other	25	29.76
**Gestational trimesters**		
1st trimester	24	28.57
2nd trimester	29	34.52
3rd trimester	31	36.91
**Stillbirth or miscarriage**		
Never	64	76.19
Once	11	13.1
Multiple	9	10.71
**Ownership of cat**	58	69.05
**Source of drinking water**		
Tap water	55	65.48
Well	18	21.43
Borehole	7	8.33
Other (Sachet)	2	2.38
Stream/river	2	2.38

### Seropositivity and associated risk factors

There were 47 (55.95%) women who tested positive for anti‐*T. gondii* antibodies; a total of 3 (3.57%) were positive for IgM, and 44 (52.38%) were positive for IgG. Seropositivity was associated with the occupation of pregnant women (*p* = 0.031) (Table 2). Women with occupations in trading (OR = 18, 95% CI 2.74–118.39, *p* = 0.003) and farming (OR = 6.75, 95% CI 1.16–39.20, *p* = 0.033) had significantly higher risk for testing seropositive (Table 3).

**TABLE 2:** Socio‐demographic factors associated with *Toxoplasma gondii* seropositivity among pregnant women attending antenatal clinic tested at Nkawie‐Toase Government Hospital, Atwima‐Nwabiagya District, Ghana, 2020.

Factors	Seropositivity		*p* value
	Positive (%)	Negative (%)	
**Age (years)**			
<20	1 (16.7)	5 (83.3)	0.083
20‐39	40 (54.05)	34 (45.95)	
≥40	3 (75)	1 (25)	
**Levels of education**			
None	9 (75)	3 (25)	0.079
Basic	32 (59.26)	22 (40.74)	
Secondary	6 (35.29)	11 (64.71)	
Tertiary	0 (0)	1 (100.0)	
**Occupation**			
Trader	16 (88.9)	2 (11.1)	0.031^a^
Other	14 (56)	11 (44)	
Farmer	9 (75)	3 (25)	
Housewife	3 (37.5)	5 (62.5)	
Caterer	2 (66.7)	1 (33.3)	
Seamstress	2 (40)	3 (60)	
Teacher	1 (33.3)	2 (66.7)	
Hairdresser	0	5 (100)	
Student	0	4 (100)	
Civil Servant	0	1 (100)	
**Gestational age**			
1st trimester	11 (45.83)	13 (54.17)	0.532
2nd trimester	17 (58.62)	12 (41.38)	
3rd trimester	19 (61.29)	12 (38.71)	
**Stillbirth or miscarriage**			
Never	33 (51.56)	31 (48.44)	0.120
Once	6 (54.55)	5 (45.45)	
Multiple	8 (88.89)	1 (11.11)	
**Ownership of cat**			
Yes	36 (62.07)	22 (37.93)	0.103
No	11 (42.31)	15 (57.69)	
**Source of drinking water**			
Tap water	33 (60)	22 (40)	0.376
Well	9 (50)	9 (50)	
Stream/river	2 (100)	0 (0)	
Borehole	2 (28.57)	5 (71.43)	
Other (Sachet)	1 (50)	1 (50)	

^a^ *p* Value statistically significant.

**TABLE 3:** Risk factors associated with *Toxoplasma gondii* seropositivity among pregnant women attending antenatal clinic tested at Nkawie‐Toase Government Hospital, Atwima‐Nwabiagya District, Ghana, 2020.

Factors	Odds ratio (95% CI)	*p* value
Age (years)		
20–39	7.19 (0.80–64.47)	0.800
≥40	Ref	
Ownership of cat	2.23 (0.87–5.72)	0.095
Stillbirth or miscarriage		
Never	Ref	
Once	1.12 (0.31–4.07)	0.855
Multiple	7.52 (0.89–63.61)	0.064
Level of education		
None	2.06 (0.50–8.49)	0.316
Basic	Ref	
Secondary	0.375 (0.12–1.16)	0.090
Tertiary	1	–
Gestational age		
1st trimester	Ref	
2nd trimester	1.67 (0.56–4.99)	0.355
3rd trimester	1.87 (0.64–5.51)	0.256
Source of drinking water		
Borehole	Ref	
Tap water	3.75 (0.67–21.07)	0.133
Well	2.5 (0.38–16.42)	0.340
Other (Sachet)	2.5 (0.10–62.60)	0.577
Stream/river	1	–
Occupation		
Caterer	Ref	
Trader	18 (2.74–118.39)	0.003^a^
Farmer	6.75 (1.16–39.20)	0.033^a^
Housewife	1.35 (0.21–8.62)	0.751
Civil servant	0.75 (0.06–9.62)	0.825
Student	1	–
Other	2.86 (0.69–11.82)	0.146

^a^ *p* values statistically significant.

## DISCUSSION

In this sample, nearly half of the pregnant women were exposed to *T. gondii* infection, and a low proportion had active infections. The seropositivity rate in this study is lower than that of 85% and 92.5% reported in Ghana's Central and Greater Accra Region, respectively [10, 32], It is, however, higher than that of 50.3% in the Ashanti [22, 27].

In our study, there was a high seropositivity rate among pregnant women involved in trading and farming. This is comparable to studies in Ethiopia where contact with soil was found as a risk factor [33, 34]. The association of trading as a risk factor for testing seropositive was also reported in Ethiopia [35]. Farming remains an important risk factor as oocysts in the soil persist with infective potential for about 12–24 months [36] or farmers are likely to be exposed to undercooked meat, salad and fruits [37].

We did not find an association between seropositivity and owning cats similar to other cross‐sectional studies from Tanzania, Nigeria and parts of Ghana, involving between 350 and 374 pregnant women [9, 38]. This is in contrast to reports from cross‐sectional studies in Taiwan and Ethiopia, involving between 201 and 426 participants, which reported that living with cats posed a significant risk of infection [39, 40, 41]. Pregnant women, however, must be made aware of the dangers of being exposed to cats or their faeces to prevent *T. gondii* infections.

It was observed in this study that the majority of the pregnant women in their third trimester were exposed to *T. gondii* infection. During pregnancy, early infection with *T. gondii* increases the risk of foetal transmission, with an increase between 60% and 81% in the third trimester [42, 43]. Infections in the first trimester can be severe, resulting in abnormalities in foetal development or abortion [25]. When infection occurs in the third trimester, 80% of new‐borns are asymptomatic [44].

Age was not found to be a determinant in testing seropositive as in data reported from elsewhere [45, 46]. In our study, a seropositivity rate of 54.05% was observed in among those aged 20–39 years, the childbearing age group. It also indicates the need to ensure the continuous education of women of childbearing age on the spread and prevention of toxoplasmosis. It is also important to note that the older the age, the longer the period of exposure to *T. gondii* and subsequently the constant level of anti‐toxoplasma IgG in the serum. This could explain why studies reported an increase in seropositivity with increasing ages [47, 48, 49].

Although other studies in Africa found a significant link between the source of drinking water and the risk of *T. gondii* infection [9, 45, 50], there was no significant evidence in this study. Seropositivity was higher in persons with lower levels of education and declines as education levels rise. This result is consistent with the findings of a study in Nigeria that reported having less education increased the probability of contracting toxoplasmosis [51].

In Ghana, there is no mandatory policy on TORCH screening among pregnant women, although there are some reports on the burden of TORCH pathogens causing infections, such as rubella virus, cytomegalovirus (CMV) and herpes simplex virus‐2 (HSV‐2) [52]. Considering the impacts of negative pregnancy outcomes, it is important to routinely screen pregnant women.

This study was limited to the period of sample collection and was based on pregnant women attending antenatal clinics.

## CONCLUSION

Half of the women attending antenatal care at Nkawie‐Toase Government Hospital in Ghana were seropositive for *T. gondii* infection with significant association with trading and farming occupations. The proportion of IgM positivity was low. Given the consequences of toxoplasmosis on pregnancy and its outcomes, it is advised that the *T. gondii* serological tests be included in the routine screening of pregnant women attending antenatal care in health facilities.

## AUTHOR CONTRIBUTIONS

*Conceptualization (support); investigation; formal analysis; writing – original draft preparation; writing – review and editing*: Seth Offei Addo. *Investigation; methodology; data curation; writing – review and editing*: Thelma Ohene Asare, Christina Arthur, Kenneth Amoateng and Kelvin Addo. *Conceptualization (lead); project administration; investigation; supervision; writing – review and editing*: John Asiedu Larbi.

## CONFLICT OF INTEREST STATEMENT

The authors declare there are no conflicts of interest in this study.

## ETHICS STATEMENT

This research was approved by the Committee on Human Research, Publications, and Ethics (CHRPE) at the Kwame Nkrumah University of Science and Technology, School of Medical Sciences and Komfo Anokye Teaching Hospital (Ref: CHRPE/AP/062/20).

## FUNDING INFORMATION

There are no funders to report for this submission.

## Data Availability

The data that supports the findings of this study are available in the supplementary material of this article.

## References

[1] Liu Q , Das Singla L , Zhou H . Vaccines against *Toxoplasma gondii*: status, challenges and future directions. Hum Vaccines Immunother. 8:1305‐1308. doi:10.4161/hv.21006 Published online 2012 PMC3579912 22906945

[2] Jones JL , Dubey JP . Foodborne toxoplasmosis. Clin Infect Dis. 2012;55(6):845‐851. doi:10.1093/cid/cis508 22618566

[3] English ED , Striepen B . The cat is out of the bag: how parasites know their hosts. PLoS Biol. 2019;17(9):6‐11. doi:10.1371/journal.pbio.3000446 PMC6748446 31487278

[4] Afonso E , Thulliez P , Gilot‐Fromont E . Local meteorological conditions, dynamics of seroconversion to *Toxoplasma gondii* in cats (*Felis catus*) and oocyst burden in a rural environment. Epidemiol Infect. 2010;138(8):1105‐1113. doi:10.1017/S0950268809991270 19961642

[5] Pappas G , Roussos N , Falagas ME . Toxoplasmosis snapshots: global status of *Toxoplasma gondii* seroprevalence and implications for pregnancy and congenital toxoplasmosis. Int J Parasitol. 2009;39(12):1385‐1394. doi:10.1016/j.ijpara.2009.04.003 19433092

[6] Tenter AM , Heckeroth AR , Weiss LM . *Toxoplasma gondii*: from animals to humans. Int J Parasitol. 2000;30(12‐13):1217‐1258. doi:10.1016/S0020-7519(00)00124-7 11113252 PMC3109627

[7] Paul E , Kiwelu I , Mmbaga B , et al. *Toxoplasma gondii* seroprevalence among pregnant women attending antenatal clinic in Northern Tanzania. Trop Med Health. 2018;46(1):1‐8. doi:10.1186/s41182-018-0122-9 30479556 PMC6245905

[8] Gelaye W , Kebede T , Hailu A . High prevalence of anti‐toxoplasma antibodies and absence of *Toxoplasma gondii* infection risk factors among pregnant women attending routine antenatal care in two Hospitals of Addis Ababa, Ethiopia. Int J Infect Dis. 2015;34:41‐45. doi:10.1016/j.ijid.2015.03.005 25759324

[9] Mwambe B , Mshana SE , Kidenya BR , et al. Sero‐prevalence and factors associated with *Toxoplasma gondii* infection among pregnant women attending antenatal care in Mwanza, Tanzania. Parasit Vectors. 2013;6(1):1‐5. doi:10.1186/1756-3305-6-222 23915834 PMC3750225

[10] Ayi I , Edu S , Apea‐Kubi K , Boamah D , Bosompem K , Edoh D . Sero‐epidemiology of toxoplasmosis amongst pregnant women in the Greater Accra region of Ghana. Ghana Med J. 2009;43(3):107‐114. doi: 10.4314/gmj.v43i3.55325 20126322 PMC2810244

[11] Halonen S , Weiss L . Toxoplasmosis. Handb Clin Neurol. 2013;114:125‐145. doi:10.1016/B978-0-444-53490-3.00008-X 23829904 PMC4157368

[12] Harker KS , Jivan E , McWhorter FY , Liu WF , Lodoen MB . Shear forces enhance *Toxoplasma gondii* tachyzoite motility on vascular endothelium. MBio. 5:e01111‐e01113.doi:10.1128/mBio.01111-13. Published online 2014. PMC3977363 24692639

[13] Furtado JM , Smith JR , Belfort R , Gattey D , Winthrop KL . Toxoplasmosis: a global threat. J Glob Infect Dis. 2011;3:281‐284. doi:10.4103/0974-777X.83536 21887062 PMC3162817

[14] Holliman RE . Congenital toxoplasmosis: prevention, screening and treatment. J Hosp Infect. 1995;30(SUPPL):179‐190. doi:10.1016/0195-6701(95)90018-7 7560949

[15] Olariu TR , Remington JS , McLeod R , Alam A , Montoya JG . Severe congenital toxoplasmosis in the United States: clinical and serologic findings in untreated infants. Pediatr Infect Dis J. 2011;30(12):1056‐1061. doi:10.1097/INF.0B013E3182343096 21956696

[16] Rostami A , Riahi SM , Contopoulos‐Ioannidis DG , et al. Acute Toxoplasma infection in pregnant women worldwide: a systematic review and meta‐analysis. PLoS Negl Trop Dis. 2019;13(10):1‐20. doi:10.1371/journal.pntd.0007807 PMC6822777 31609966

[17] Sitoe SPBL , Rafael B , Meireles LR , de Andrade HF , Thompson R . Preliminary report of HIV and *Toxoplasma gondii* occurrence in pregnant women from Mozambique. Rev Inst Med Trop Sao Paulo. 2010;52(6):291‐295. doi:10.1590/S0036-46652010000600002 21225211

[18] Domingos A , Ito LS , Coelho E , Lúcio JM , Matida LH , Ramos AN . Seroprevalence of *Toxoplasma gondii* igg antibody in HIV/AIDS‐infected individuals in Maputo, Mozambique. Rev Saude Publica. 2013;47(5):890‐896. doi:10.1590/S0034-8910.2013047004661 24626493

[19] Linguissi LSG , Nagalo BM , Bisseye C , et al. Seroprevalence of toxoplasmosis and rubella in pregnant women attending antenatal private clinic at Ouagadougou, Burkina Faso. Asian Pac J Trop Med. 2012;5(10):810‐813. doi:10.1016/S1995-7645(12)60148-5 23043921

[20] Akinbami A , Adewunmi A , Rabiu K , et al. Seroprevalence of *Toxoplasma gondii* antibodies amongst pregnant women at the Lagos State University Teaching Hospital, Nigeria. Niger Postgrad Med J. 2010;17(2):164‐167. Accessed January 18, 2022. https://pubmed.ncbi.nlm.nih.gov/20539334/ 20539334

[21] De Paschale M , Ceriani C , Cerulli T , et al. Antenatal screening for *Toxoplasma gondii*, Cytomegalovirus, rubella and Treponema pallidum infections in northern Benin. Trop Med Int Health. 2014;19(6):743‐746. doi:10.1111/tmi.12296 24612218

[22] Ayi I , Sowah AOK , Blay EA , Suzuki T , Ohta N , Ayeh‐Kumi PF . *Toxoplasma gondii* infections among pregnant women, children and HIV‐seropositive persons in Accra, Ghana. Trop Med Health. 2016;44(1):1‐8. doi:10.1186/s41182-016-0018-5 27433136 PMC4940749

[23] Sefah‐Boakye J , Frimpong E , Dompreh H , Turpin A . Seroprevalence of *Toxoplasma gondii* Infection among Pregnant Women in the Ashanti Region Of Ghana. Int J Microbiol Adv Immunol. 2016;4(1):70‐74. 10.19070/2329-9967-1600013

[24] Arthur‐mensah R , Blay E , Ayi I , Larbi J , Suzuki T , Nobuo O . Effectiveness of SP‐IPTp for Malaria and Evidence for the Need of *T. gondii* Infection Preventive Policy during Pregnancy in Ghana. J Infect Dis Epidemiol. 2016;2(3). 10.23937/2474-3658/1510018

[25] Singh B , Debrah LB , Acheampong G , Debrah AY . Seroprevalence and risk factors of *toxoplasma gondii* infection among pregnant women in Kumasi: a cross‐sectional study at a district‐level hospital, Ghana. Infect Dis Obstet Gynecol. 2021;2021. doi:10.1155/2021/6670219 PMC8041552 33883871

[26] Kwofie KD , Ghansah A , Osei JHN , et al. Indication of Risk of Mother‐to‐Child *Toxoplasma gondii* Transmission in the Greater Accra Region of Ghana. Matern Child Health J. 2016;20(12):2581‐2588. doi:10.1007/S10995-016-2084-Z 27465060

[27] Agordzo SK , Badu K , Addo MG , et al. Seroprevalence, risk factors and impact of *Toxoplasma gondii* infection on haematological parameters in the Ashanti region of Ghana: a cross‐sectional study. AAS Open Res. 2019;2:166. 10.12688/aasopenres.13022.1 32734139 PMC7369427

[28] Calderaro A , Peruzzi S , Piccolo G , et al. Laboratory diagnosis of *Toxoplasma gondii* infection. Int J Med Sci. 2009;6(3):135‐136. doi:10.7150/ijms.6.135 19319234 PMC2659488

[29] Montoya JG , Liesenfeld O . Toxoplasmosis. Elsevier; 2004:1965‐1976. doi:10.1016/S0140-6736(04)16412-X 15194258

[30] Ayawine A , Ae‐Ngibise KA . Determinants of exclusive breastfeeding: a study of two sub‐districts in the Atwima Nwabiagya District of Ghana. Pan Afr Med J. 2015;22:248. 10.11604/pamj.2015.22.248.6904 26958111 PMC4764318

[31] Lima‐Oliveira G , Lippi G , Salvagno GL , Picheth G , Guidi GC . Laboratory diagnostics and quality of blood collection. J Med Biochem. 2015;34(3):288‐294. doi:10.2478/jomb-2014-0043 28356839 PMC4922344

[32] Abu EK , Boampong JN , Ayi I , et al. Infection risk factors associated with seropositivity for *Toxoplasma gondii* in a population‐based study in the Central Region, Ghana. Epidemiol Infect. 2015;143(9):1904‐1912. doi:10.1017/S0950268814002957 25373611 PMC9507263

[33] Gebremedhin EZ , Abebe AH , Tessema TS , et al. Seroepidemiology of *Toxoplasma gondii* infection in women of child‐bearing age in central Ethiopia. BMC Infect Dis. 2013;13(1):1. doi:10.1186/1471-2334-13-101 23442946 PMC3598201

[34] Abamecha F , Awel H . Seroprevalence and risk factors of *Toxoplasma gondii* infection in pregnant women following antenatal care at Mizan Aman General Hospital, Bench Maji Zone (BMZ), Ethiopia. BMC Infect Dis. 2016;16(1):1‐8. doi:10.1186/s12879-016-1806-6 27585863 PMC5007994

[35] Adugna B , Tarekegn ZS , Damtie D , et al. Seroepidemiology of *Toxoplasma gondii* among pregnant women attending antenatal care in northwest Ethiopia. Infect Drug Resist. 2021;14:1295‐1303. doi:10.2147/IDR.S299106 33883909 PMC8053702

[36] Dubey JP . Sources of *Toxoplasma gondii* infection in pregnancy. Until rates of congenital toxoplasmosis fall, control measures are essential. BMJ. 2000;321(7254):127‐128. doi:10.1136/BMJ.321.7254.127 10894674 PMC1118145

[37] Iddawela D , Vithana SMP , Ratnayake C . Seroprevalence of toxoplasmosis and risk factors of *Toxoplasma gondii* infection among pregnant women in Sri Lanka: a cross sectional study. BMC Public Health. 2017;17(1):1‐6. doi:10.1186/s12889-017-4941-0 29202747 PMC5716377

[38] Ishaku B , Ajogi I , Umoh JU , Lawal I , Randawa AJ . Seroprevalence and Risk Factors for Toxoplasma gondii Infection among Antenatal Women in Zaria. Res J Med Sci. 2009;4(2):483‐488.

[39] Zemene E , Yewhalaw D , Abera S , Belay T , Samuel A , Zeynudin A . Seroprevalence of *Toxoplasma gondii* and associated risk factors among pregnant women in Jimma town, Southwestern Ethiopia. BMC Infect Dis. 2012;12(1):1‐6. doi:10.1186/1471-2334-12-337 23216887 PMC3519766

[40] Lin YL , Liao YS , Liao LR , Chen FN , Kuo HM , He S . Seroprevalence and sources of Toxoplasma infection among indigenous and immigrant pregnant women in Taiwan. Parasitol Res. 2008;103(1):67‐74. doi:10.1007/s00436-008-0928-1 18327612

[41] Awoke K , Nibret E , Munshea A . Sero‐prevalence and associated risk factors of *Toxoplasma gondii* infection among pregnant women attending antenatal care at Felege Hiwot Referral Hospital, northwest Ethiopia. Asian Pac J Trop Med. 2015;8(7):549‐554. doi:10.1016/j.apjtm.2015.06.014 26276286

[42] Robert‐Gangneux F , Dardé ML . Epidemiology of and diagnostic strategies for toxoplasmosis. Clin Microbiol Rev. 2012:264‐296. doi:10.1128/CMR.05013-11. Published online. 22491772 PMC3346298

[43] Moncada PA , Montoya JG . Toxoplasmosis in the fetus and newborn: an update on prevalence, diagnosis and treatment. Expert Rev Anti Infect Ther. 2014;10(7):815‐828. doi:10.1586/ERI.12.58 22943404

[44] Hassaneina F , Shehata A . Rapid immunochromatographic test (RDT) versus ELISA technique for diagnosing toxoplasmosis among individuals with mental disabilities. Int J Innov Res Dev. 2018;7(6):61‐66. 10.24940/ijird/2018/v7/i6/JUN18058

[45] Murebwayire E , Njanaake K , Ngabonziza JCS , Njunwa KJ , Jaoko W . Seroprevalence and risk factors of *Toxoplasma gondii* infection among pregnant women attending antenatal care in Kigali, Rwanda. Tanzan J Health Res. doi:10.4314/thrb.v19i1.2. Published online 2017.

[46] Andiappan H , Nissapatorn V , Sawangjaroen N , et al. Knowledge and practice on Toxoplasma infection in pregnant women from Malaysia, Philippines, and Thailand. Front Microbiol. 2014;5(JUN):291. doi:10.3389/fmicb.2014.00291 24966855 PMC4052801

[47] Khademi SZ , Ghaffarifar F , Dalimi A , Davoodian P , Abdoli A . Prevalence and risk factors of *Toxoplasma gondii* infection among pregnant women in Hormozgan province, south of Iran. Iran J Parasitol. 2019;14(1):167‐173. 10.18502/ijpa.v14i1.732 31123482 PMC6511600

[48] Onduru OG , Aboud S . Prevalence and risk factors for typical signs and symptoms of toxoplasmosis in children born to at risk pregnant women attending prenatal care in Temeke district, Tanzania. Sci Afr. 2021;11:e00690. doi:10.1016/j.sciaf.2020.e00690

[49] Al‐Harthi SA , Jamjoom MB , Ghazi HO . Seroprevalence of *Toxoplasma gondii* among pregnant women in Slovakia. Umm Al‐Qura Univ J Sci Med Eng. 2006;18(2):217‐227.

[50] Alsammani MA . Sero‐epidemiology and risk factors for *Toxoplasma gondii* among pregnant women in Arab and African countries. J Parasit Dis. 40:569‐579. doi:10.1007/s12639-014-0558-8. Published online 2016. PMC4996171 27605750

[51] Ishaku BS , Ajogi I , Umoh JU , Lawal I , Randawa AJ . Seroprevalence and risk factors for *Toxoplasma Gondii* infection among antenatal women in Zaria, Nigeria. Res J Med Med Sci. 2009;4(2):483‐488.

[52] Kwofie TB , Ayensu F , Mutocheluh M , et al. Seroprevalence of rubella virus, cytomegalovirus and herpes simplex virus‐2 among pregnant women at the Komfo Anokye Teaching Hospital, Ghana. J Public Heal Dev Ctries. 2015;1(2):56‐63. http://www.jphdc.org/

